# Abdominal aortic angiosarcoma following endovascular aneurysm repair: case report and analysis of existing literature

**DOI:** 10.3389/fsurg.2026.1972877

**Published:** 2026-10-14

**Authors:** Loes H. Willems, Marcus Steinbach, Daniel Pink, Thomas Herold, Alexander Meyer, Roger Wahba

**Affiliations:** 1 Clinic of Vascular Surgery, Helios Klinikum Berlin-Buch, Berlin, Germany; 2 Clinic of General, Visceral and Oncological Surgery, Helios Klinikum Berlin-Buch, Berlin, Germany; 3 Department of Oncology and Palliative Care, Helios Klinikum Berlin-Buch, Berlin, Germany; 4 Department of Internal Medicine, University Hospital Greifswald, Greifswald, Germany; 5 Clinic of Diagnostic and Interventional Radiology, Helios Klinikum Berlin-Buch, Berlin, Germany; 6 Medical School Berlin, Berlin, Germany

**Keywords:** abdominal aortic aneurysm, aortic angiosarcoma, case report, endovascular aneurysm repair, multimodal therapy

## Abstract

Abdominal aortic angiosarcoma following endovascular aneurysm repair (EVAR) is an exceptionally rare and aggressive malignancy that may mimic common EVAR-related complications. We report the case of a 72-year-old man who presented with progressive lower back pain and was initially suspected of having a graft infection. Imaging revealed a periaortic mass involving a previously treated abdominal aortic aneurysm. Biopsy confirmed aortic angiosarcoma. No regional lymph node or distant metastases were identified. The patient underwent neoadjuvant chemotherapy with gemcitabine and docetaxel, followed by radiotherapy and surgical resection of the abdominal aorta and EVAR graft with aortic reconstruction. Histopathological examination demonstrated complete pathological response without evidence of residual angiosarcoma. Our literature review identified only 21 reported cases of abdominal aortic angiosarcoma following EVAR, including the present case, underscoring the exceptional rarity of this entity and the limited evidence available to guide diagnosis and treatment. This case highlights the importance of considering aortic angiosarcoma in patients with persistent symptoms and atypical periaortic findings after EVAR and suggests that early biopsy and a multidisciplinary, multimodal treatment approach may improve outcomes despite the generally poor prognosis associated with this disease.

## Introduction

Angiosarcomas are rare malignant neoplasms derived from endothelial cells, accounting for about 2% of all soft-tissue sarcomas, with an annual incidence of one case per million persons (1, 2). Angiosarcomas may arise in any soft tissue. Primary angiosarcoma of the aortic wall is an extremely rare and aggressive entity that is generally characterized by poor survival outcomes (3). Early diagnosis and treatment are essential, however, they remain challenging due to nonspecific symptoms (4). Few cases of aortic angiosarcoma following endovascular aneurysm repair (EVAR) have been described in literature. In this setting particularly, angiosarcomas are frequently misdiagnosed as EVAR-related complications, such as graft infection or endoleak, resulting in delays in diagnosis and treatment (5–7). Diagnostic modalities that may raise suspicion of angiosarcoma include computed tomography (CT), magnetic resonance imaging (MRI), and positron emission tomography (PET)-CT. Imaging features suggestive of aortic angiosarcoma include aortic wall irregularity, protrusive appearance, higher FDG uptake, and contrast enhancement on MRI (8). Because of the rarity of the disease, treatment is not standardized. Limited evidence suggests that a combination of surgical resection, aortic reconstruction, and chemoradiotherapy may be the preferred treatment approach (3). We present an unusual and particularly rare case of abdominal aortic angiosarcoma following EVAR, in which multimodal treatment resulted in complete pathological response of the tumor.

## Case report

A 72-year-old man presented to the emergency department with progressive lower back pain over the preceding six months, radiating to both flanks. There was no history of fever, chills, or night sweats. His medical history included hyperlipidemia, hypertension, benign prostatic hyperplasia, renal cysts, and previous crossectomy of the left great saphenous vein. In addition, the patient had undergone endovascular aneurysm repair (EVAR, Gore Excluder) for a 5.5 cm abdominal aortic aneurysm five years before in another institution. Follow-up consisted of annual computed tomography angiography (CTA). Two and five years after the primary procedure, type II endoleaks originating from lumbar arteries were diagnosed and treated with coil embolization, the most recent intervention having been performed approximately two years before presentation to the emergency department. In the preceding six months, the patient had been treated with analgesics for MRI-confirmed lumbar spine osteoarthritis. Medications included metamizole, hydromorphone, and fentanyl. Despite chronic opioid therapy, the lower back pain progressively worsened. Physical examination revealed no abnormalities, and femoral pulses were palpable bilaterally. Laboratory investigations demonstrated an elevated C-reactive protein (CRP) level of 68 mg/L, leukocytosis (11.5 Gpt/L), and a negative procalcitonin (PCT) result. Retrospectively, the most recent follow-up CTA, performed approximately one year earlier, had revealed soft-tissue thickening of the dorsal aortic wall measuring 1.7 cm. The patient was admitted to the vascular surgery ward with a suspected graft infection. A Positron Emissions Tomography- (PET)-CT was performed and confirmed a retroperitoneal mass involving the previously treated abdominal aortic aneurysm, demonstrating increased glucose uptake and raising suspicion of either an infectious or a malignant process (Figure 1). Empirical treatment with piperacillin/tazobactam was initiated but did not result in symptom resolution or normalization of laboratory parameters. Blood cultures remained negative. Therefore, an infectious etiology of the soft-tissue thickening appeared unlikely. During hospitalization, the patient developed asymptomatic grade 2 left-sided hydronephrosis, which was treated with ureteral stent placement. A CT-guided core needle biopsy of the aortic wall was inconclusive. A second biopsy revealed a high-grade malignant non-small-cell tumor with glandular-like structures, a predominantly solid and trabecular growth pattern, slit-like spaces with micropapillary structures and intracytoplasmic lumen formation, as well as strong expression of ERG, CD31, and c-MYC, with a proliferation index of approximately 70%, consistent with angiosarcoma. CT imaging demonstrated no evidence of lymphogenic or distant metastases, and the multidisciplinary tumor board classified the tumor as cX N0 M0. Following multidisciplinary discussion at the institutional tumor board, the patient received neoadjuvant chemotherapy consisting of six cycles of gemcitabine and docetaxel, followed by neoadjuvant radiotherapy to a total dose of 50 Gy (2-Gy fractions administered once daily, five days per week). Repeated follow-up CT scans demonstrated stable disease, with no progression of the abdominal aortic angiosarcoma and no evidence of lymphogenic or distant metastases. The repeat PET-CT scan revealed complete resolution of the glucose hypermetabolism that had been present prior to treatment. After completion of neoadjuvant therapy, median laparotomy was performed, with resection of the abdominal aorta and the EVAR graft, followed by aortic reconstruction using a silver-coated aortobi-iliac Dacron graft (20 × 11 mm) (Figure 2). The estimated blood loss was 3 L and the patient received 6 units of packed red blood cells, 6 units of fresh frozen plasma. The patient was transferred to the intensive care unit until postoperative day three. A total of six units of packed red blood cells and five units of fresh frozen plasma were transfused, as well as 2 g of fibrinogen, and 1 g of tranexamic acid. No surgical complications occurred. The patient was discharged from the surgical ward on postoperative day nine. Pathological examination of the resected aorta and EVAR graft revealed no histological or immunohistochemical evidence of residual angiosarcoma. Informed consent was obtained from the patient.

**Figure 1:**
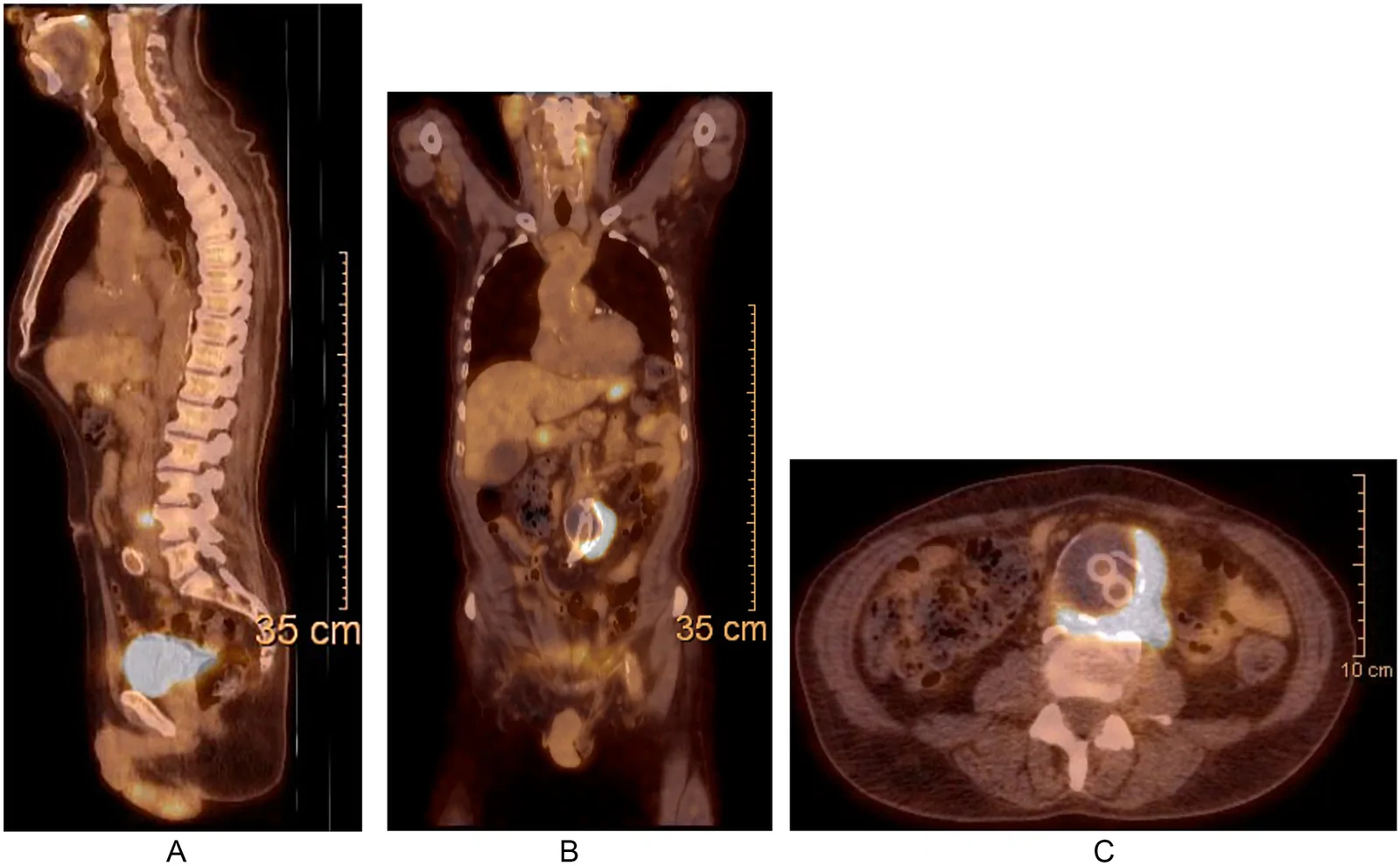
Positron emission tomography computed tomography demonstrating metabolically active soft-tissue thickening surrounding the distal abdominal aorta at the level of the aortic endograft bifurcation, corresponding to the subsequently diagnosed abdominal aortic angiosarcoma, in coronal **(A)**, sagittal **(B)**, and axial **(C)** planes.

**Figure 2:**
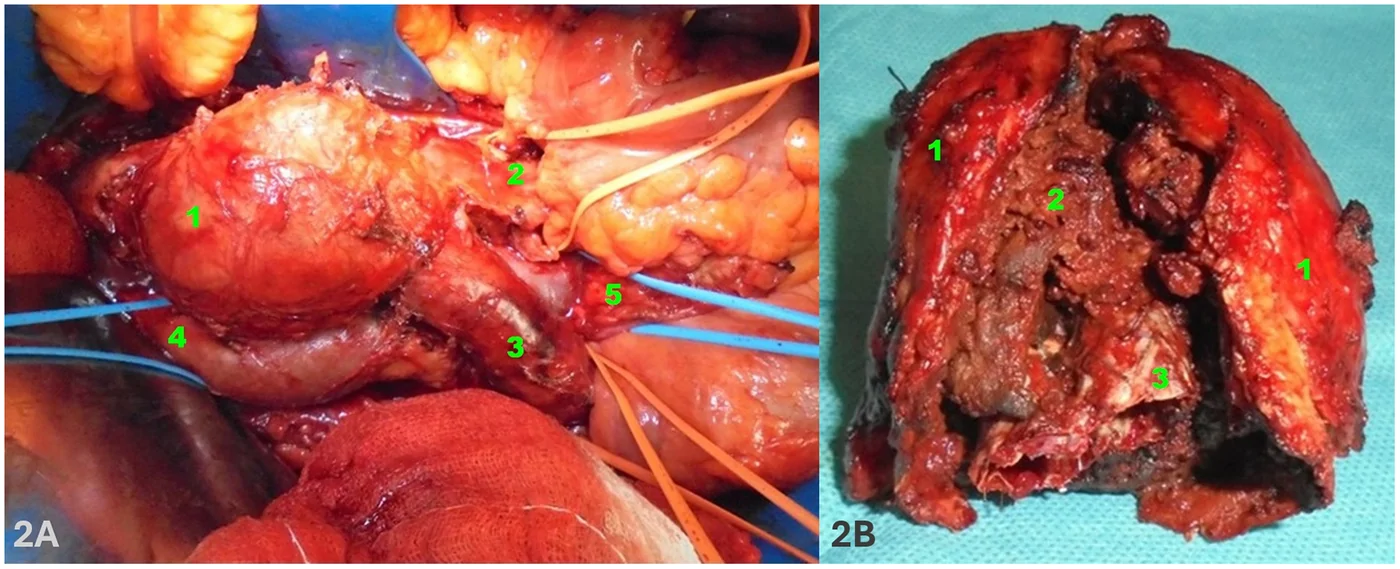
Intraoperative photographs of **(A)** the exposed abdominal aortic bifurcation, showing tumorous thickening of the distal aorta (1). Both common iliac arteries (2,3), the inferior vena cava (4), and the right common iliac vein (5) are encircled with vessel loops. **(B)** The *en bloc* resected abdominal aortic aneurysm, showing tumorous thickening of the distal aorta (1), intra-aneurysmal mural thrombus (2), and the endovascular aneurysm repair (EVAR) graft (3).

## Discussion

Aortic angiosarcomas are extremely rare soft-tissue malignancies, arising from the malignant transformation of endothelial cells within the aortic wall (1). Clinical presentation is generally nonspecific, posing substantial diagnostic challenges. In a systematic review, Kordzadeh et al. described the epidemiology and clinical presentation of aortic angiosarcomas and identified lower limb claudication (25%), abdominal or back pain (22%), and weight loss (11%) as the most frequently reported symptoms. Concerning angiosarcoma of the abdominal aortic wall specifically, Leriche syndrome was radiologically confirmed in approximately 60% of reported cases. Furthermore, the median age at presentation was 67 years, sex distribution was equal, and the median survival following diagnosis was 180 days (4). The diagnosis of abdominal aortic angiosarcoma after EVAR may be particularly challenging, as the most common symptom, lower limb claudication, may occur late or may be absent due to mechanical preservation of the aortic lumen by the endograft, thereby preventing tumor-related occlusion and the development of Leriche syndrome. In addition, the differential diagnosis is more complex in patients with previous EVAR, as EVAR-related complications must also be considered and are considerably more prevalent. These include graft infection, endoleak, and late aneurysm rupture, with reported incidences of 1.4% (9), 11.7% (10), and 2.5% (11), respectively, within five years following EVAR. In the present case, the patient presented exclusively with lower back pain. Weight loss and other B symptoms were absent. Lower limb claudication and Leriche syndrome were excluded both clinically, as femoral pulses were palpable bilaterally, and radiologically, as no evidence of aortoiliac stenosis or occlusion was identified.

We performed an extensive literature review and identified 18 publications reporting a total of 20 cases of abdominal aortic angiosarcoma developing during follow-up after EVAR for abdominal aortic aneurysm (Supplementary Table 1) (5–7, 12–25). Including the present case, a total of 21 cases were available for analysis. The median time to the development of angiosarcoma was 7 [range 3,5–10] years. Clinical presentation was described for 16 patients (5–7, 12–16, 18–22, 24, 25). The most frequently reported symptoms were abdominal pain (*n* = 9), back pain (*n* = 9), and inguinal or leg pain (*n* = 5), although only two patients presented with typical intermittent claudication. Fever and weight loss were reported in seven cases, and fatigue in four cases. Less common presentations included gastrointestinal bleeding and other gastrointestinal complaints (*n* = 2). The initial suspected diagnoses included graft infection, endoleak, aortoenteric fistula, aortitis, and spondylodiscitis in 15, 9, 4, 2, and 2 of the 21 cases, respectively. Treatment data were available for 19 patients and included surgery (*n* = 13), chemotherapy (*n* = 8), and radiotherapy (*n* = 4) (5–7, 12–16, 18–25). Overall prognosis was poor. Fifteen patients died shortly after diagnosis (5–7, 12–21, 24, 25), while outcome data were unavailable for three patients (17, 23). Only three patients survived primary treatment. Among these, one patient achieved complete remission following chemoradiotherapy, although follow-up was not reported (14). Another patient was initially diagnosed with non-metastatic disease and underwent complete surgical (R0) resection of the tumor followed by adjuvant chemotherapy, however, metastatic disease developed one year after surgery, and the patient died 24 months postoperatively (22). The third patient was the present case. At the time of writing, the patient remains disease-free 4 months after R0 surgical resection.

An important consideration in the present case is whether the lesion treated by EVAR five years earlier could already have represented a primary abdominal aortic angiosarcoma rather than an abdominal aortic aneurysm. Although this possibility cannot be completely excluded, we consider it highly unlikely. Primary aortic angiosarcoma is characterized by rapid local progression and early metastatic spread. In a systematic review including 122 patients with primary aortic sarcoma, the median survival was only 180 days despite individualized multimodal treatment, including surgery, chemotherapy, and radiotherapy (4). In contrast, our patient remained clinically stable for five years after EVAR without radiological or clinical evidence of progressive malignant disease.

An equally important question is whether earlier radiological recognition would have been possible during routine EVAR surveillance. Retrospectively, the follow-up CTA performed approximately one year before diagnosis already demonstrated soft-tissue thickening of the dorsal aortic wall. Previous research has described several imaging features that may raise suspicion for aortic angiosarcoma, including a pedunculated mass, an atypical filling defect, extension beyond the vascular wall, and contrast enhancement on MRI reflecting lesion vascularization (8). Although the CTA findings in the present case were nonspecific, further evaluation of otherwise unexplained periaortic soft-tissue thickening using MRI for tissue characterization and PET-CT to assess metabolic activity may facilitate earlier diagnosis.

Beyond the clinical and radiological diagnostic challenges, histopathological confirmation of aortic angiosarcoma may also be particularly demanding. Because of its location within or adjacent to the aortic wall and its vascular nature, obtaining representative tissue can be difficult and carries procedural risks. In the present case, the first CT-guided core needle biopsy was inconclusive, and the diagnosis was established only after repeat biopsy. In the surgically resected aorta, no histological or immunohistochemical evidence of residual angiosarcoma was identified following neoadjuvant chemoradiotherapy, providing pathological evidence of a complete response. Consequently, the diagnosis ultimately relied on a relatively small biopsy specimen, making histopathological characterization of the entire tumor impossible.

## Conclusion

Abdominal aortic angiosarcoma following EVAR is an exceptionally rare and aggressive malignancy that may mimic common EVAR-related complications, resulting in delayed diagnosis. Consideration of malignancy in patients with persistent symptoms and atypical periaortic findings after EVAR is of utmost importance, particularly when conventional treatment fails and imaging findings remain unexplained. Clinicians should maintain a low threshold for further diagnostic evaluation when unexplained periaortic soft-tissue abnormalities are identified during EVAR follow-up to facilitate earlier recognition. This case demonstrates that early biopsy, multidisciplinary evaluation, and aggressive multimodal treatment can result in pathological complete response, despite the generally poor prognosis associated with this disease.

## Data Availability

The original contributions presented in the study are included in the article/Supplementary Material, further inquiries can be directed to the corresponding author.
