# Supplementary material for: Abdominal aortic angiosarcoma following endovascular aneurysm repair: case report and analysis of existing literature
Source: Front Surg. 2026 Oct 14;13:1972877. doi: 10.3389/fsurg.2026.1972877 (PMC13713609; doi:10.3389/fsurg.2026.1972877)
Supplement: Supplementary file 1 [file Table1.docx]

Supplementary table 1: overview of the identified 18 publications reporting a total of 20 cases of abdominal aortic angiosarcoma

| Case | Study | Time to development of angiosarcoma [Years] | Clinical presentation | Initial suspected diagnoses | Treatment | Survival |
| --- | --- | --- | --- | --- | --- | --- |
| 1 | Brendle C^12^ | 8 | Fatique, abdominal pain | Graft infect, spondylodiscitis | Surgery | No |
| 2 | Cleret de Langavant B^5^ | 4 | Back/inguinal pain, claudication, endograft stenosis | Mural thrombus, endoleak, graft infection | Surgery, chemotherapy | No |
| 3 | Derouane F^13^ | 3,5 | Claudication, weight loss | Stent stenosis, endoleak, graft infection | Chemotherapy | No |
| 4 | Fenton J^6^ | 6 | Back pain, weight loss | Endoleak, graft infection | Surgery, chemotherapy radiotherapy | No |
| 5 | Garg N^7^ | * | Back/inguinal pain, weight loss | Graft infection | Surgery | No |
| 6 | Kamran M, 1^14^ | 8 | Weight loss, abdominal pain | endoleak | Chemotherapy, radiotherapy | Yes |
| 7 | Kamran M, 2^14^ | * | Abdominal pain, bilious emesis | Endoleak, aortic rupture | None | No |
| 8 | Koussayer S^15^ | 8 | Weight loss, constipation, fever, increased urination, back pain | graft infection, aortitis | Chemotherapy, radiotherapy | No |
| 9 | Milite D^16^ | 7 | Abdominal pain, fatique, fever | Endoleak, graft infection | Surgery | No |
| 10 | Momand G^2^ | 10 | Abdominal/leg pain, fever, malaise | Graft infection | Surgery | No |
| 11 | Pecceu S, 1^17^ | * | * | Graft infection | * | * |
| 12 | Pecceu S, 2^17^ | * | * | Graft infection | * | * |
| 13 | Presacco S^18^ | 5 | Back/leg pain | Vertebral neoplasm, osteonecrosis, spondylitis | Surgery | No |
| 14 | Rota M^19^ | 8 | Back pain, fever | Endoleak, graft infection | Surgery | No |
| 15 | Schmehl J^20^ | 7 | Fatigue, weight loss, abdominal pain | Endoleak, arteriitis, graft infection | Surgery | No |
| 16 | Stewart B^21^ | 9 | Gastrointestinal bleeding, fever | Aortocolonic fistula | Surgery | No |
| 17 | Umscheid TW^22^ | 4 | Abdominal pain, melena, weight loss | Aortoduodenal fistula, graft infection | Surgery, chemotherapy | Yes† |
| 18 | Walker K^23^ | * | * | Endoleak | Chemotherapy | * |
| 19 | Whittington EA^24^ | 10 | Abdominal/back pain, fever | Polymyalgia rheumatica, endoleak | Surgery | No |
| 20 | Yu PC^25^ | 4 | Fever, back/abdominal pain | Endoleak, graft infection | Surgery | No |

* = not reported. †primary survival, recurrence after 1 year, died of metastases after 2 year
